# In-depth characterization of food and environmental microbiomes across different meat processing plants

**DOI:** 10.1186/s40168-024-01856-3

**Published:** 2024-10-15

**Authors:** Coral Barcenilla, José F. Cobo-Díaz, Alba Puente, Vincenzo Valentino, Francesca De Filippis, Danilo Ercolini, Niccolò Carlino, Federica Pinto, Nicola Segata, Miguel Prieto, Mercedes López, Avelino Alvarez-Ordóñez

**Affiliations:** 1 Department of Food Hygiene and Technology and Institute of Food Science and Technology, Universidad de León, 24071 León, Spain; 2 Department of Agricultural Sciences, University of Naples Federico II, 80055 Portici, Italy; 3 Task Force On Microbiome Studies, University of Naples Federico II, 80138 Naples, Italy; 4 Department of Cellular, Computational and Integrative Biology, University of Trento, 38123 Trento, Italy

**Keywords:** Meat, Metagenomics, Microbiome, Resistome, Virulome, Resident microbiota, Food processing environments

## Abstract

**Background:**

Processing environments can be an important source of pathogenic and spoilage microorganisms that cross contaminate meat and meat products. The aim of this study was to characterize the microbiome of raw materials, processing environments and end products from 19 facilities producing different meat products.

**Results:**

The taxonomic profiles of the microbial communities evolved along processing, from raw materials to end products, suggesting that food contact (FC) surfaces play an important role in modulating the microbiome of final products. Some species persisted with the highest relative abundance in raw materials, food processing environments and/or in the final product, including species from the genera *Pseudomonas*, *Staphylococcus*, *Brochothrix*, *Acinetobacter* and *Psychrobacter*. Processing environments showed a very diverse core microbiota, partially shared with the products. *Pseudomonas fragi* and *Pseudomonas sp*. Lz4W (in all sample and facility types) and *Brochothrix thermosphacta*, *Psychrobacter sp.* and *Psychrobacter* sp. P11F6 (in raw materials, FC surfaces and end products) were prominent members of the core microbiota for all facilities, while *Latilactobacillus sakei* was found as a dominant species exclusively in end products from the facilities producing fermented sausages. Processing environments showed a higher amount of antimicrobial resistance genes and virulence factors than raw materials and end products. One thousand four hundred twenty-one medium/high-quality metagenome-assembled genomes (MAGs) were reconstructed. Of these, 274 high-quality MAGs (completeness > 90%) corresponded to 210 putative new species, mostly found in processing environments. For two relevant taxa in meat curing and fermentation processes (*S. equorum* and *L. sakei*, respectively), phylogenetic variation was observed associated with the specific processing facility under study, which suggests that specific strains of these taxa may be selected in different meat processing plants, likely contributing to the peculiar sensorial traits of the end products produced in them.

**Conclusions:**

Overall, our findings provide the most detailed metagenomics-based perspective up to now of the microbes that thrive in meat, meat products and associated environments and open avenues for future research activities to better understand the microbiome functionality and potential contribution to meat quality and safety.

Video Abstract

**Supplementary Information:**

The online version contains supplementary material available at 10.1186/s40168-024-01856-3.

## Background

Raw meat has optimal characteristics that allow the development of bacteria, such as large nutrient availability, high water activity (a_w_) and slightly acid pH [1]. Among the different bacteria that can grow in meat, special attention should be given to particular pathogenic and spoilage species which can lead to public health issues, important quality losses and changes in the physico-chemical and organoleptic properties of meat and meat products, with associated economic losses for food businesses [2]. Meat and meat products are common vehicles for the transmission of the main pathogens responsible for foodborne infections. Moreover, the spoilage of meat and meat products caused by the proliferation of microorganisms related to gas production, slime and softening, discoloration and/or luminescence is also a cause of major concern [3]. It is estimated that meat or meat products represent 21% of all the food wasted in Europe and North America [4]. Bacterial activity is the most common cause of meat spoilage, which is influenced by multiple factors (e.g. product composition, manufacture conditions, storage temperature, packaging). Under aerobic conditions, *Pseudomonas*, *Acinetobacter*, *Brochothrix*, *Shewanella* and *Aeromonas* species are common meat spoilers, whereas in anaerobic conditions, *Weissella*, *Leuconostoc*, *Bacillus* and *Clostridium* species are more relevant [3]. Lactic acid bacteria (LAB), mainly *Carnobacterium*, *Lactococcus* and members of the former *Lactobacillus*, are also commonly identified as meat spoilers in anaerobic conditions [5, 6].

Certain ubiquitous microorganisms are commonly found in meat processing plants and have the ability to form biofilms and survive disinfection procedures, acting as a source for further contamination of meat and meat products [7, 8]. Although the muscle of a healthy animal is sterile, the carcass obtained after slaughter will easily be contaminated by microorganisms present on the slaughterhouse surfaces, equipment (e.g. tables and knives) and environments (e.g. the air in the refrigeration chambers). Moreover, some operations pose a risk of cross-contamination of carcasses with the animals’ endogenous skin and gut microbes (e.g. dehairing, evisceration). Carcasses are then transported to meat processing plants, where meat is exposed to other environments (i.e., contact and non-contact surfaces), which can also be a source of new microbes. Thus, the acquired microbiota will evolve all along the meat processing chain, considering also that each room within the processing facilities can have different resident microbial communities [9–11]. A meta-analysis performed by Xu et al. [12] suggested that processing facilities can represent a niche for persistence of autochthonous bacteria, influenced by selection and dispersal processes.

The bacteria colonizing meat processing environments are frequently resistant to antimicrobials, posing an additional risk to human health. The use of antibiotics in livestock is an important aspect to take into account, as it has promoted the spread of antimicrobial-resistant bacteria in farms, slaughterhouses and meat processing plants [13].

In order to extend the shelf life of meat and enhance its safety, different strategies that inhibit or retard the development of pathogenic and spoilage microorganisms are adopted by meat processors. Meat ageing, curing and fermentation are processes primarily or secondarily pursuing this objective, and all of them are characterized by the important modifications in the physico-chemical parameters of raw meat they entail, which act as selective pressure shaping microbiome successions along shelf life [14]. Altogether, the changes occurring during these processes help to obtain a safe and high-quality product [15, 16].

Traditionally, the study of microbial communities was based on culturing bacteria with the subsequent characterization of isolates, but culture-based approaches cannot provide a complete picture of the true microbial diversity within a sample, its genetic repertoire or functional potential [17]. On the contrary, the use of whole metagenome sequencing (WMS) can allow to better understand dynamic changes in the microbiome and characterize some particular genes of interest, like virulence factors and antimicrobial resistance genes [7, 10]. The aim of this study was to provide an in-depth characterization, across different meat processing chains, of the microbiome of fresh, dry-aged, cured and fermented meat products and their associated processing environments, though the analysis by WMS of 220 samples of raw materials, processing environments and end products from 19 meat companies.

## Methods

### Sampling and samples pre-processing

Nineteen meat processing facilities located in *León* (Spain), producing cured beef (*n* = 6), dry-aged beef (*n* = 2), fermented pork sausages (*n* = 7) and fresh pork (or mixed pork-beef) meat products (*n* = 4), were sampled (Table S1). The facilities were visited on a single occasion before the start of the morning shift. Thus, samples were taken before production started and a few hours after the completion of the cleaning and disinfection procedures. A total of 220 samples were taken and processed. This included 135 samples from food processing environments (67 food contact (FC) surfaces and 68 non-food contact (NFC) surfaces), 22 samples of raw meat before processing (raw materials), 38 samples of end product after processing or ripening/curing/ageing, and, in the case of fermented sausages, 13 samples of the meat batter before stuffing and 12 samples of the sausages after stuffing and before ripening (Table S1).

FC and NFC surfaces were sampled following an improved sampling procedure for microbiome analysis in food processing environments recently developed in the frame of the EU project MASTER [18]. In each facility, FC and NFC samples were taken from different rooms throughout the processing line, which includes processing, cold, smoking, maturation/ripening and/or packaging rooms. Briefly, 5 Whirl–Pak Hydrated PolyProbe swabs (Whirl–Pak, Wisconsin, US) were collected and pooled in order to increase the microbial loads recovered from each sample type included in the study. The choice of polyurethane swabs instead of those of cellulose-derived materials was made to improve microbial recovery and avoid contamination with non-microbial DNA. Each swab was employed to recover the microbial cells from a ⋍1 m^2^ surface. In those cases where a surface of 1 m^2^ could not be sampled, one unit was swabbed (e.g. one knife, one drain). For raw materials (in facilities processing fresh meat products, dry-aged meat and cured meat) and end products (in dry-aged meat and cured meat industries) the surface of meat cuts was sampled before and after processing and/or ageing/curing following the same approach previously described for surfaces, also obtaining pools of 5 swabs for each sample to be sequenced. In the case of companies producing fermented pork sausages, > 20 g of meat batter (in duplicate), two sausages before ripening and two sausages after ripening were taken for direct analysis without swabbing. This same approach was also followed for end fresh meat products (fresh sausages or minced meat after processing). The end products corresponded to the batch of production of the same day that raw materials and processing environments were sampled. Thus, they were collected after the ripening/curing/ageing process, meaning some weeks later, except for dry-aged and cured meat products, which generally were subjected to very long ageing/curing periods. Final products from a different batch of production were collected the same day of sampling in those cases. Duplicate samples were taken for each raw material and final product.

Gloves, disposable coats, caps, shoes and facial masks were used during sampling, and gloves were changed between samples to avoid cross-contamination. Each sampling was performed following the food chain production flow to avoid cross-contamination of the products or environments, and samples were kept refrigerated in a portable cooler with ice packs until sample processing in the laboratory, which was performed within the next 24 h after sampling.

Samples labelled as C01 and F02 correspond to the same industry but different production lines, one for a cured meat product (*cecina*) and other for a dry fermented sausage (*chorizo*). Both products are ripened in the same room, but there is no physical contact between them. For the other industries, the sampled product was the only one being processed during the sampling day, but in some cases the operator produced other products in the same line (on different days along the week). There is no possible physical contact between raw materials and final products for any of the sampled industries, since the industries follow hygienic zoning principles and have separated storage rooms for raw materials and final products, to avoid possible cross-contamination events. Staff movement was also generally regulated to avoid cross-contamination, with the exception of some artisanal small producers.

Sample manipulation at the laboratory was performed in a laminar flow hood, using gloves. The harvesting of the microbial biomass from the swab samples taken from surfaces or meat was performed by adding 10 mL of 1X sterile phosphate buffered saline (PBS; Sigma-Aldrich, Missouri, US) to the sampling bag containing the pool of 5 swabs, followed by homogenization in a stomacher (IUL Instruments, Barcelona, Spain) (175 rpm for 2 min), the recovery of 10 mL of homogenized liquid and its centrifugation at 5000 × *g* for 5 min. The tubes with the cell pellets were stored in an ultra-freezer at − 80 °C. In the case of meat batter and pork sausage samples, 10 g of sample was weighed and homogenized with 90 mL of PBS using a stomacher (175 rpm for 2 min). Afterwards, the homogenate was centrifuged at 5000 × *g* for 15 min at room temperature and any fat layer was removed. Cell pellets were resuspended with 10 mL of PBS, and the previous washing step was repeated three times. The cell pellets obtained after the final wash were kept at − 80 °C until DNA extraction.

### DNA extraction and sequencing

DNA isolation was performed using a recently developed protocol [18] based on the DNeasy PowerSoil Pro kit (QIAGEN, Hilden, Germany) with some modifications, which include (i) the use of QIAamp UCP MinElute spin columns (QIAGEN) instead of standard spin columns; (ii) the addition of 600 μL of isopropanol plus 600 μL of Solution CD3 during the DNA binding step; (iii) the use of a customized wash buffer CD5, obtained by mixing 500 μL of CD5 and 333 μL of ethanol 100%; and (iv) the elution in a final volume of 20 μL [18]. DNA concentration was quantified using the Qubit High Sensitive quantification kit (Thermo Fisher Scientific, Massachusetts, US).

The libraries for Illumina NovaSeq metagenomic sequencing were prepared with the Nextera DNA Flex Library Prep kit (Illumina, California, US) following the manufacturer’s instructions. Metagenomic libraries were multiplexed using dual indexing and sequenced for 150 bp paired-end reads (average of 7.5 GB/sample) on a NovaSeq 6000 Sequencing System (Macrogen, Seoul, South Korea).

### Bioinformatic analyses

Raw reads were quality-filtered using the pre-processing pipeline available at [19]. Firstly, sequencing adapters, reads of low quality (Phred score < 20), short reads (< 75 bp), and reads with more than 2 ambiguous nucleotides were removed using Trim Galore (v0.6.6) [20]. Contaminant DNA was identified using Bowtie2 v2.2.9 [21] with *–sensitive-local* parameter, removing reads aligning to the reference genomes phiX174 and the GRCh38.p13 human genome (GCF_000001405.39).

Before taxonomic assignment, the corresponding meat host genome (assembly GCF_002263795 for cow, *Bos taurus*, and GCF_000003025 for pig, *Sus scrofa*) was also employed to detect and remove contaminant non-microbial DNA. Taxonomic assignment of filtered reads was done using Kraken2 v2.0.8-beta [22] with a modified version of the PlusPF database, updated on the 12th January 2024 (available at [23]) using default parameters. The modifications on PlusPF consisted in the addition of representative genomes for those species found at MAG level missed in the PlusPF database or, for those species with no representative genome or classified as *Genus_name* sp. *strain_code*, where *strain_code* is an alphanumeric code, the genome employed for MAGs taxonomical assignment by GTDB-tk. Selected genomes (Table S2) were downloaded from National Center for Biotechnology Information (NCBI) using *FTP Path* column in *prokaryotes.txt* file from NCBI ftp repository (>ftp://ftp.ncbi.nlm.nih.gov/genomes/GENOME_REPORTS/prokaryotes.txt) and downloading the*_genomic.fna.gz file*, which is the genome in*.fasta* format. Genome downloading was done manually on the NCBI web (https://www.ncbi.nlm.nih.gov/) for those genomes which missed *FTP path* on *prokaryotes.txt* file (Table S2). Downloaded genomes were decompressed by *gzip -d* command and added to the kraken2 database following the instructions available at (https://github.com/DerrickWood/kraken2/blob/master/docs/MANUAL.markdown). For those genomes with taxonomy not correctly “read” during *kraken2-build –build* step (indicated in *unmaped.txt* file within folder database) the “|kraken2:taxid|*taxid_code” *was added to each header of the genome, being *taxid_code* the taxid code from NCBI (indicated on kraken2:taxid column at Table S2), before the genome addition to the kraken2 database. Furthermore, both *Bos taurus* and *Sus scrofa* reference genomes employed for host-removal by bowtie were also added to the database, as an extra safety check to remove host reads. Finally, Bracken [24] was employed to improve species abundance estimations, transforming the kraken2 database to bracken format indicating a 150-bp length (*bracken-build -d PlusPF_modified/ -t 64 -k 35 -l 150*), which was also indicated during bracken run (*-r 150*).

The alpha-diversity values of species richness and the Simpson diversity index were calculated using the R package *vegan*.

The core microbiota was calculated taking those species present in > 90% of samples and with more than 1% relative abundance in at least 10% of samples. Samples were grouped according to industry type and sample category. *ComplexUpset* v. 1.3.3 [25] R-package was used to create an upset plot representing the core microbiota.

For resistome and virulome analysis, filtered reads were aligned versus the ResFinder database [26] and Virulence Factor DataBase [27], respectively, using bowtie2 [21] with *–very-sensitive –end-to-end* parameters. Obtained sam files were filtered by an in-house ruby script (*count_reads.rb*) (https://github.com/SegataLab/MASTER-WP5-pipelines/blob/master/07-AMR_virulence_genes/count_reads.rb), which removes the gene over-estimation occurring when forward and reverse reads are aligned with the same gene. The obtained counts matrix was processed by R-scripts to calculate the counts per million reads (CPM) adding a “bacterial marker” modification according to the formula:

$$\text{CPM}=(\text{target}-\text{genes}-\text{reads}*{10}^{6}*\text{Bacterial Markers alignment})/\text{total reads}$$

where CPM is the total counts per million reads value for each gene; target-genes-reads are the number of reads that match with the target genes; “Bacterial Markers alignment” is the value obtained from viromeQC [28], using *–minlen 0 –minqual 0* parameters; and “total reads” is the total number of reads obtained on each sample. The “Bacterial Markers alignment” value indicates the proportion of bacterial DNA on a metagenomic fastq file, thus applying this parameter on the equation we remove those reads not assigned to bacterial taxa.

Moreover, as reads were 150 bp length and some antimicrobial resistance genes (ARG) larger than 150 bp differ by one or a few single-nucleotide polymorphisms (SNPs), genes from the ResFinder database were clustered at 90% identity using CDHIT [29] with default values, and ARG classified according to this clustering (Genefam column at Table S3).

For the virulome, the abundance of biofilm-related genes, adherence-related genes and exotoxin-related genes included in the Virulence Factor Database [27] was represented on plots with *ggplot2* v. 3.3.3. Additionally, the number of reads assigned to *Staphylococcus aureus* exotoxins and to genes related to *Escherichia coli* pathotypes (Table S4), selected according to Koutsoumanis et al. [30], was also analysed.

Using a single-sample metagenomics assembly approach, reads were assembled into contigs using MEGAHIT v1.1.124 [31] with default parameters. Contigs longer than 1000 nt were then binned using MetaBAT2 v2.12.125 [32] with parameters *–maxP 95 –minS 60 –maxEdges 200 –unbinned –seed 0*. Quality control of the MAGs was performed using CheckM v1.0.726 [33] with default parameters. Only high-quality (completeness > 90%, contamination < 5%) and medium-quality (completeness between 50 and 90%, contamination < 5%) MAGs were kept for further analysis, according to parameters proposed by Pasolli et al. [34]. Taxonomic assignment of MAGs was performed by GT-DBTk v2.1.1 [35] using the *classify_wf* command. The MAGs obtained were employed for the calculation of Average Nucleotide Identity (ANI) values using dRep v2.6.2 [36]. The *Mdb.txt* output file obtained from dRep was transformed into a distance matrix, which was employed for the construction of a phylogenetic tree plot by *ggtree* R-package, using the UPGMA clustering algorithm (hclust method = "average").

Assembly-free strain-level population genomics analyses were performed for those species of special interest using the StrainPhlan pipeline [37]. Before StrainPhlan analysis, MetaPhlAn3 [38] was run versus the mpa_v30_CHOCOPhlAn_201901 database for each sample using the paired filtered reads and –*bowtie2out* option to obtain the alignment files in*.sam* format. The *sample2makers.py* script was used to recover the markers files for each sample, and *extract_markers.py* to extract clade-specific markers for each species under investigation. Phylogenetic trees were plotted by *ggtree* R-package, using the UPGMA clustering algorithm (*hclust* method = "average") and the *RaxML_bestTree* output file from StrainPhlan analysis.

All analyses were carried out using R v4.0.2 [39].

Fastq files are available at the Sequence Read Archive of the NCBI, under the bioproject accession number PRJNA997800.

### Statistical analyses

Comparisons between multiple groups of samples for taxonomy were performed by using the Kruskal Wallis rank sum test and the post hoc Pairwise Wilcoxon sum rank test, with *p*-values adjusted through the Benjamini and Hochberg method [40].

The *compare_means* function, from *ggpubr* R-package [41], was employed to include statistically significant differences on boxplot figures, which were plotted by ggplot2 R-package [42]. For beta-diversity analysis, principal coordinates analysis using Bray–Curtis dissimilarity was used by the *vegdist* function while within-group dispersion was evaluated through the *betadisper* function. Variable effects on sample dissimilarities were determined by permutational multivariate analysis of variance (PERMANOVA) using distance matrices with the *adonis* function. The *vegdist*, *betadisper* and *adonis* functions are available in *vegan* R-package [43].

## Results

### Surfaces contain a more diverse microbiome and some FC environments show a taxonomic profile close to that of end products

**Fig. 1:**
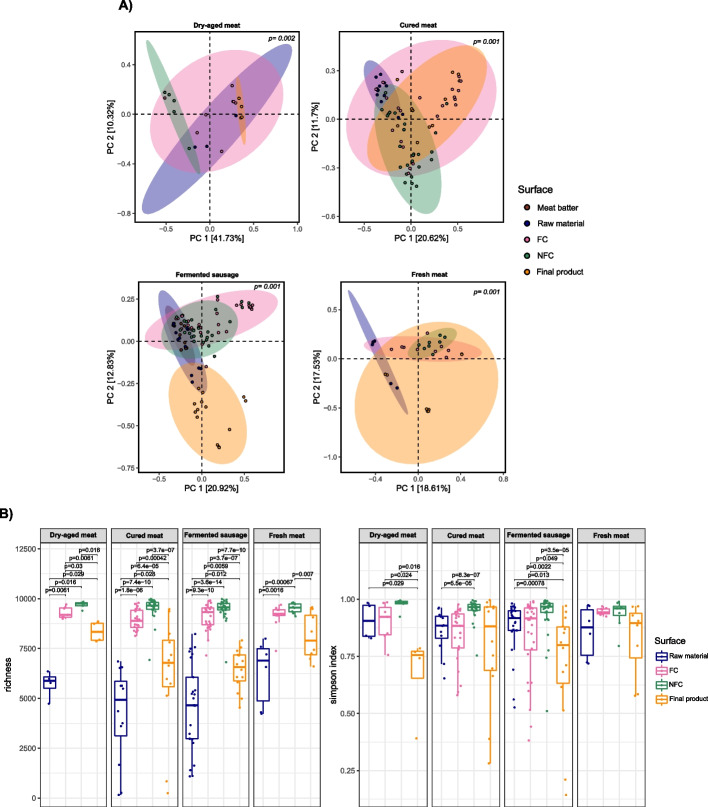
Alpha- and beta-diversity. **A** Principal coordinates analysis (PCoA) based on Bray–Curtis dissimilarity metrics of the bacterial communities of the different samples taken in each meat industry sector. **B** Alpha diversity indices of meat before and after ripening/processing and food contact and non-food contact surfaces for each meat industry sector

As shown in the Bray–Curtis beta diversity distance matrix principal coordinates analysis, the sample type variable had a significant influence on the taxonomic profile of samples (adonis, *p* ≤ 0.001) and explained 52.05, 32.32, 33.75 and 34.14% of the variation observed in dry-aged meat, cured meat, fermented sausages and fresh meat product industries, respectively (Fig. 1A). Ordination analyses grouped separately samples from raw material or meat batter and final product, especially for cured meat and fermented sausages facilities, with FC surfaces being grouped in the case of cured meats closer than raw material samples to end product samples (Fig. 1A).

There were also statistically significant differences in the taxonomic profiles of microbial communities among meat sectors for raw materials, FC surfaces and final products (adonis *p* < 0.05), but not for NFC surfaces (*p* = 0.068) (Fig. S1). The sector type explained a 32.94, 42.22 and 45.07% of the variation observed in raw materials, FC surfaces and final products, respectively.

Generally, there was an evident pattern of richness differences among sample types, with NFC surfaces having the highest richness followed by FC surfaces, final products and raw materials (Fig. 1B). In fresh meat product facilities, the differences in richness were only statistically significant between surfaces and raw materials, and NFC surfaces and final products (Fig. 1B). Simpson indices were, in general, close to 1, especially in NFC and FC surfaces, which indicates a high diversity. However, some samples had Simpson indices < 0.5, belonging principally to five final product and two FC surface samples, due to the dominant role of one species over the rest. Dry-aged beef showed similar Simpson indices across all (NFC and FC) surface samples. Final products had significantly less Simpson index values than other sample types for dry-aged meat and fermented sausages facilities (Fig. 1B).

### The main bacterial taxa identified differ by sample category and industry type

**Fig. 2:**
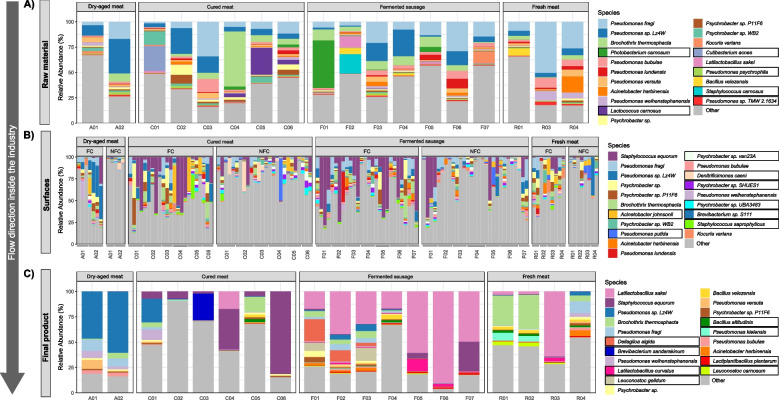
Bacterial taxonomic composition in the meat production systems. Relative abundance (%) of the 20 most abundant species in **A** raw materials or intermediate products before ripening, **B** food contact and non-food contact surfaces and **C** final product samples from each facility. Species highlighted in boxes persist among the most abundant ones in at least 2 of these sample categories (raw materials, surfaces and final products). In fermented sausages facilities, the raw material category refers to meat batter and sausages before ripening

In most cases, the microbiome of raw material samples was dominated by a few bacterial taxa, with one unique species standing out with high relative abundances, ranging from 26.3% (R04) to 54.7% (C05). *Pseudomonas fragi* was the dominant species for raw materials in some companies, including in 1 cured meat, 4 fermented sausage and 3 fresh meat products facilities (Fig. 2A). *Pseudomonas* sp. *Lz4W* was predominant in raw materials from the 2 dry-aged meat, 1 cured meat and 1 fermented sausage facilities, but commonly present in the rest of meat processing plants. *Brochothrix thermosphacta* and *Photobacterium carnosusm* were predominant species in raw materials from 1 cured meat and 1 fermented sausages plant, respectively (with no significant differences in its abundance among industry sectors) (Fig. 2A; Fig. S2).

There were some taxonomic differences when comparing raw materials coming from beef or pork meat origin (Fig. S3A). Various *Psychrobacter* sp. were significantly (*p* ≤ 0.001) more abundant in beef than in pork raw materials. Conversely, *P. carnosum* was significantly more abundant in pork than in beef raw materials (*p* < 0.05), probably due to its high abundance in one facility. *Latilactobacillus sakei*, *Bacillus velezensis* and *Staphylococcus carnosus*, although not very abundant, were significantly more abundant in pork raw meat. Moreover, some species possibly associated with safety concerns were more frequently represented in pork than in beef raw materials (*p* < 0.05), including *Enterobacter hormaechei*, *Klebsiella pneumoniae*, *Salmonella enterica*, *Acinetobacter baumannii*, *Enterobacter cloacae* and *Yersinia enterocolitica* (Fig. S3B).

Regarding environmental sources, processing surfaces were dominated by *Staphylococcus equorum*, followed by *P. fragi* and *Pseudomonas* sp. *Lz4W*, together with some *Psychrobacter* species (Fig. 2B; Fig. S2). Food contact (FC) surfaces were mainly dominated by *S. equorum* in cured meat and fermented sausage facilities, and *Pseudomonas* sp. *Lz4W* in dry-aged meat facilities (Fig. 2B; Fig. S2). The microbial communities of NFC surfaces were much more complex, containing a wide range of low abundant species with cumulative relative abundances of 92.1 ± 5.1%, 76.0 ± 13.7%, 72.5 ± 25.4% and 78.4 ± 15.3% for dry-aged meat, cured meat, fermented sausages and fresh meat products industries, respectively (Fig. 2B; Fig. S2). No statistically significant differences were observed among meat sectors in the taxonomic composition of NFC surfaces.

Additional bacterial species became for the first time as most abundant in the final products (Fig. 2C; Fig. S3). In particular, *L. sakei* (*p* < 0.001), *Dellaglioa algida* (*p* < 0.05), *Leuconostoc gelidum* (*p* < 0.01) and *Latilactobacillus curvatus* (*p* < 0.05) appeared as predominant in fermented sausages. *L. sakei* was the main species in final fermented sausages at 6 of the 7 facilities (44% average relative abundance), while *D. algida* was the main taxa in the other facility (22% relative abundance) (Fig. 2C; Fig. S3). *S. equorum* was the dominant species in final product from 3 cured meat facilities, with *Brevibacterium sandarakinum*, *Pseudomonas* sp. Lz4W and *B. thermosphacta* being the main species in other individual final product from cured meat facilities (Fig. 2C; Fig. S2). In general, the high relative abundance of *S. equorum* in surfaces is also maintained in final products of all cured meat and one fermented sausage facilities (Fig. 2C; Fig. S2). Some taxa with very low abundance (or absence) in raw materials, like *S. equorum*, *L. sakei* and *B. sandarakinum*, had a statistically significant increase in relative abundance along processing and ripening of fermented sausages and/or cured meat, while others with high abundance in raw materials or initial stages of production, such as *P. carnosum* and *Lactococcus carnosum*, showed sharp decreases in relative abundance along shelf life or ripening (*p* < 0.05) (Fig. 2C). Generally, the microbial composition of dry-aged meat was characterized by the high abundance of *Pseudomonas* sp. Lz4W, with 46 and 60% of relative abundance for each of the two samples analysed. Finally, in fresh meat product facilities, *B. thermosphacta* or *L. sakei*, were the dominant species in the final products of three fresh meat-producing facilities, while the fourth facility had a variety of species (Fig. 2C; Fig. S3).

### Some taxa potentially associated with safety concerns are frequently detected, mainly in raw materials and end products

**Fig. 3:**
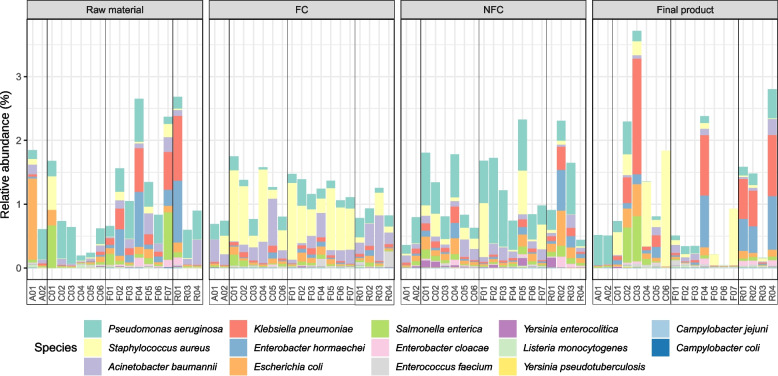
Relative abundances of those bacterial species potentially associated with safety concerns. The relative abundance found in the four types of samples analysed (raw material, food contact, non-food contact and final products) are represented. The legend presents the species detected sorted by decreasing order of relative abundance. In fermented sausages facilities, the raw material category refers to meat batter and sausages before ripening

The evaluation of the presence of bacterial species potentially linked to safety concerns in the food system, either due to their pathogenic potential or to their frequent association with antimicrobial resistance, showed that most of them were present only at a low relative abundance, ranging from mean values of 1.9^−6^ to 1.8%, for *Yersinia pseudotuberculosis* and *S. aureus*, respectively (Fig. 3). *P. aeruginosa* was the most abundant species associated with safety concerns detected, especially in NFC surfaces, with an average relative abundance of 0.3%. *S. aureus*, with 0.25% average relative abundance, was mainly detected in FC surfaces, although it was highly present in the final products of some processing facilities (Fig. 3). Raw materials from fermented sausages processing plants (meat batter and sausages just after stuffing) presented generally a higher abundance of these taxa than those from the other industry types, likely due to the pre-processing these samples had (e.g. mincing).

### Processing environments have a very diverse core microbiota, partially shared with end products

**Fig. 4:**
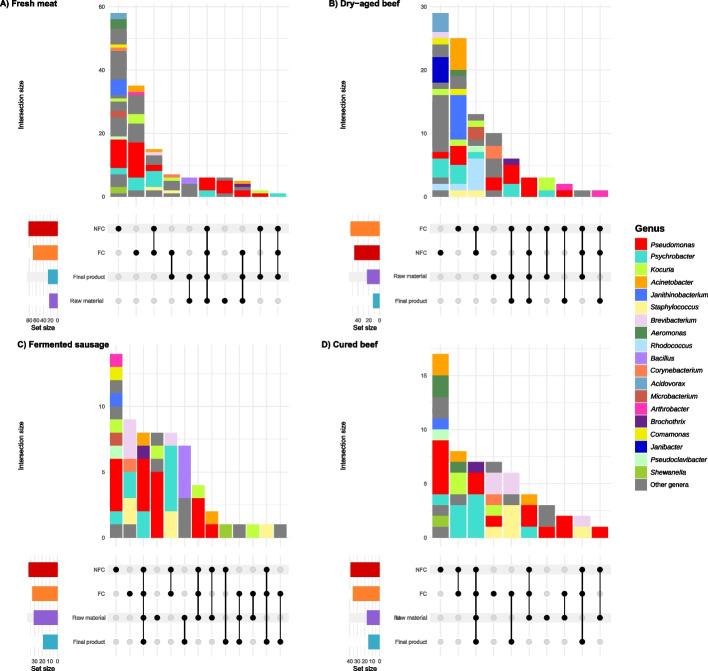
Core microbiota shared among sample types in each sector. **A** Fresh meat product industries, **B** dry-aged meat-producing industries, **C** fermented sausage-producing industries and **D** cured meat-producing industries. The core microbiota is calculated as those species present on at least 90% of the samples and with relative abundance higher than 1% on at least 10% of the samples. All species corresponding to the same genus were coloured together. Only the 20 most relevant genera are represented

The core microbiota was calculated in this study, for each sample category and industry type, considering the species present in at least 90% of the samples of each category and with relative abundance higher than 1% in at least 10% of the samples. Regarding sector type, the most diverse core microbiota was present in fresh meat product industries, with a total of 141 different bacterial species, followed by dry-aged meat companies (*n* = 93), fermented sausages processing plants (*n* = 65) and cured meat facilities (*n* = 57) (Fig. 4). Only 6, 3, 8 and 7 members of the core microbiota were shared among all the 4 sample categories (raw materials/intermediate product before ripening; end products; FC and NFC surfaces) for fresh meat products (4 *Pseudomonas* species, including *Pseudomonas* sp*. Lz4W*, *P. fragi*, *P. lundensis*, *P. bubulae*; and *2 Psychrobacter* species), dry-aged beef (3 *Pseudomonas* species, including *Pseudomonas* sp. *Lz4W*, P*. fragi* and *P. weihenstephanensis*), fermented sausages (4 *Pseudomonas* species, including *Pseudomonas* sp. *Lz4W*, *P. fragi*, *P. bubulae* and *P. versuta*; 2 *Psychrobacter* species; *Acinetobacter harbinensis*; and *B. thermosphacta*) and cured meat (4 *Psychrobacter* species; *P. fragi; Pseudomonas sp.* Lz4W; and *B. thermosphacta*) industries, respectively (Fig. 4). Interestingly, *P. fragi* and *Pseudomonas sp*. Lz4W were the only species included in the core microbiota of all of the sample groups. Several species constituted the core microbiota of both final products and FC surfaces of the facilities: *Corynebacterium variabile*, *Kocuria varians* and *Staphylococcus saprophyticus* in fresh meat product facilities (Fig. 4A); *Arthrobacter alpinus* and *P. lundensis* in dry-aged beef facilities (Fig. 4B); *Levilactobacillus brevis* in fermented sausages facilities (Fig. 4C); and *Brevibacterium sandarakinum*, *Brevibacterium antiquum*, *S. aureus*, *S. saprophyticus* and *S. casei* in cured meat facilities (Fig. 4D). However, between NFC and final products there was only 3 species in common, which were *Pseudomonas kielensis* and *Kocuria palustris* in fresh meat products facilities and *Shewanella baltica* in fermented sausage facilities (Fig. 4). Among FC and NFC, a maximum of 15 species were shared in fresh meat product facilities and a minimum of 8 in cured and fermented sausage facilities (Fig. 4). It is also remarkable that FC (with between 7 and 35 species) and NFC (with between 14 and 58 species) surfaces, in all industry types, were the sample types which hold the most diverse core microbiota (Fig. 4).

Regardless of the meat industry sector, several bacterial species were consistently shared between samples of the same type: a total of 137, 114, 62 and 54 species were found in the core microbiota for NFC surfaces, FC surfaces, final products and raw materials, respectively (Fig. S4). FC surfaces were the sample types where the highest number of species in the core microbiota was shared among the four industry types, with 16 species, mainly from the genera *Pseudomonas* and *Psychrobacter* (Fig. S4), while in final products only 2 *Pseudomonas*, 2 *Psychrobacter* and *B. thermosphacta* were shared among all four meat production facility types. Remarkably, *P. fragi* and *Pseudomonas sp. Lz4W* were shared among all industry types as a member of the core microbiota for all sample types; and *B. thermosphacta*, *Psychrobacter* sp. P11F6 and *Psychrobacter sp*. for all except NFC surfaces (Fig. S4).

### Processing environments are a reservoir of antimicrobial resistance and virulence factor genes

**Fig. 5:**
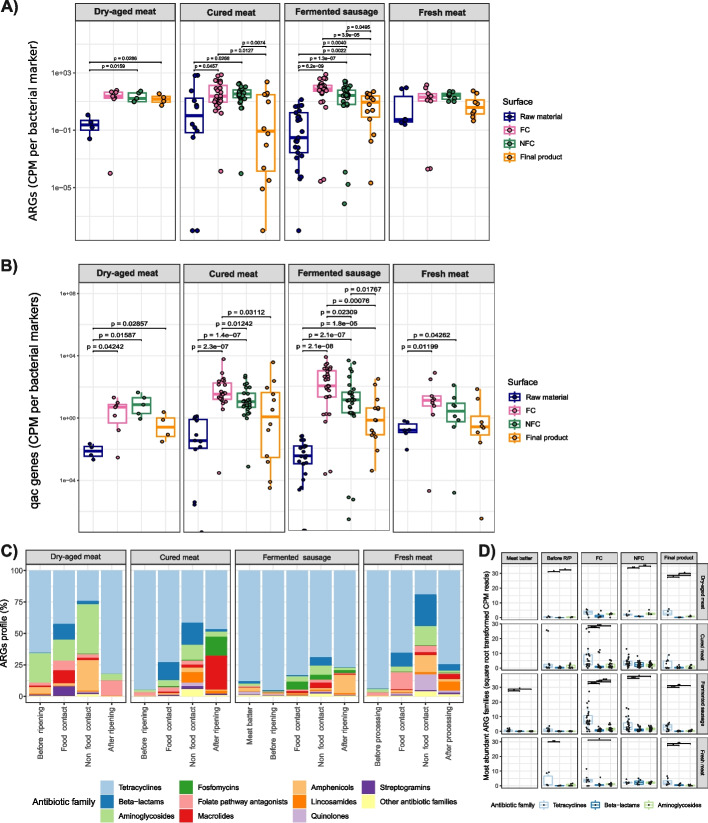
Resistome analysis. **A** Amount of antimicrobial resistance genes, expressed in CPM per bacterial marker, across the different sample types grouped by meat industry sector. **B** Amount of *qac* genes, expressed in CPM per bacterial marker, across the different sample types grouped by meat industry sector. **C** Profile of ARG detected among the different sample types and meat production sectors. Average values of the percentage of ARG identified as associated with resistance to different antimicrobial classes are presented for each sample type and meat industry sector. **D** The three most abundant ARG families, i.e. tetracyclines, beta-lactams and aminoglycosides, represented as count per million reads (square root transformed) separated by industry sector and sample type

Significantly higher amounts of ARG were found in final products, FC and NFC surfaces than in the products before ripening in facilities producing fermented sausages. On the contrary, higher levels of ARG were found in FC and NFC surfaces compared with raw materials and end products in cured meat facilities, as well as in FC surfaces of dry-aged meat plants compared to the raw materials (Fig. 5A). Furthermore, no significant differences on the amounts of ARG were observed among industry types for any of the sample categories analysed, except for the higher values found on FC surfaces in fermented sausage plants as compared to dry-aged meat and fresh meat product facilities (Fig. S5).

Similar patterns were observed for *qac* genes, which confer resistance to quaternary ammonium compounds. Again, higher amounts were observed in final products and facility surfaces, with higher levels on FC surfaces in cured meats and fermented sausage-producing facilities than on FC surfaces from dry-aged meat plants (Fig. 5B and Fig. S6). Additionally, FC surfaces presented higher levels of *qac* genes than NFC surfaces in both cured meats and fermented sausage-producing facilities (Fig. 5B). FC from dry-aged meat facilities had lower amounts of *qac* genes than FC from other industry types, while samples of intermediate products before ripening/processing from fermented sausage industries had lower values than raw materials from other food industry types (Fig. S6).

Generally, among the ARG identified, the vast majority were associated with resistance to tetracyclines, which represented from 18.9 to 95% of all ARG detected (Fig. 5C). However, ARG associated with resistance to many other antimicrobials, such as beta-lactams, aminoglycosides, fosfomycins, folate pathway antagonists, macrolides, amphenicols, lincosamides, quinolones or streptogramins, were also frequently detected. Tetracycline ARG were especially dominant in the ARG profile of fermented sausage-producing facilities and in raw materials from cured and fresh meat product processing plants (Fig. 5C). However, there were no significant differences in their CPM reads among the four industry types (Fig. S5). There were significantly more beta-lactam ARG in cured meat and folate pathway antagonists ARG in dry-aged meat than in fermented sausage industries and more fosfomycin ARG in cured than in dry-aged meat industries. In addition, in dry-aged meat-producing facilities, there were significantly more aminoglycosides and amphenicols ARG in NFC surfaces than in meat before processing and final products (Fig. 5C). In cured meat-producing facilities, beta-lactam ARG were significantly more abundant in FC and NFC than in meat before and after ripening, and FC contained significantly higher amounts of beta-lactam ARG than NFC. Fosfomycin ARG were also significantly more abundant in final products than in the meat before ripening. In fermented sausage-producing facilities, there were no significant differences between meat batter and sausages before ripening, and most ARG families were significantly less abundant in these samples than in FC and NFC surfaces. Amphenicols ARG were significantly more abundant in final products. In fresh meat product facilities, beta-lactams, aminoglycosides and amphenicols ARG were statistically more abundant in NFC surfaces than in the rest of samples (Fig. 5C).

**Fig. 6:**
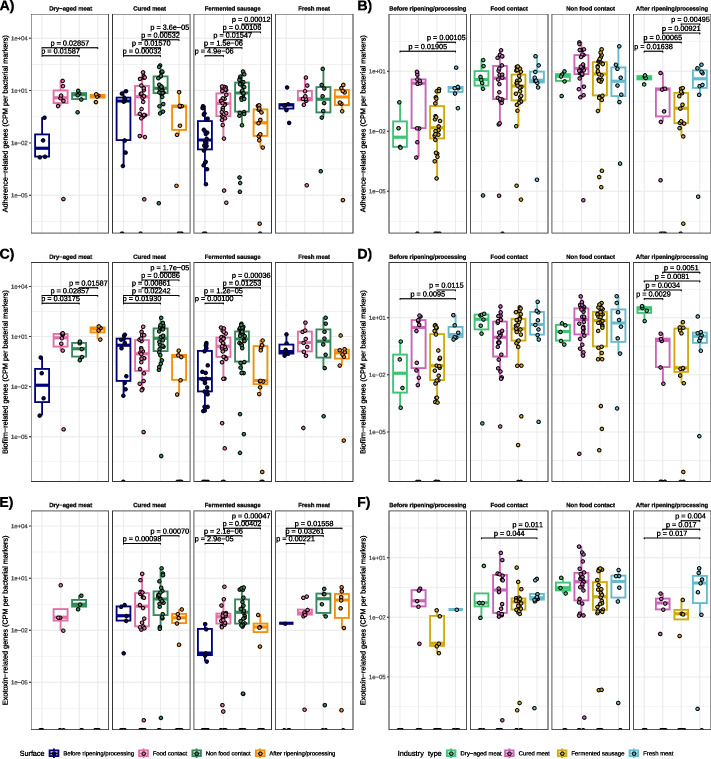
Virulence factors found in the four meat processing plants among different types of surfaces. Amount of virulence factor genes related to **A**,**B** biofilm formation, **C**,**D** adherence and **E**,**F** exotoxin production

Although no statistically significant differences were found on the amount of virulence factor (VF) genes associated with biofilm formation among meat industry sectors, FC and NFC surfaces contained higher levels than raw materials/intermediate products before ripening and final products for cured meats and fermented sausage-producing plants (Fig. 6A, B). On the other hand, final products from dry-aged meat-producing industries had significantly higher abundance of biofilm-related VF genes than raw materials, while no differences among sample types were found in fresh meat product facilities (Fig. 6A, B).

Regarding VF adherence-related genes, samples from fermented sausages facilities had significantly lower amounts than cured meat and fresh meat product facilities. Similar to biofilm-related VF genes, adherence-related VF genes were significantly more abundant on FC and NFC surfaces than on raw materials/intermediate products and final products in cured meat and fermented sausage-producing facilities, while final products, FC and NFC surfaces had higher levels than raw materials in dry-aged meat-producing facilities (Fig. 6C, D). Moreover, for exotoxin-related VF genes, the lowest counts were detected in fermented sausage companies and the highest in cured meat facilities, with a general increase of such VF genes on FC and NFC surfaces compared to raw materials/intermediate products and final products (Fig. 6E, F).

All the ARG and virulence factor genes detected in our analyses are listed in Table S5 and Table S6, respectively. Remarkably, some *S. aureus* enterotoxin genes and genes associated with *E. coli* pathotypes were detected, although with very low occurrence and abundance. Only 2 genes associated with *E. coli* pathotypes were found, *cylA*, on 2 final products, and *eae*, on 2 FC surfaces (Fig. S7A). Regarding *S. aureus*, a total of 15 exotoxin genes were found, mainly in FC surfaces, with a FC sample from a fermented sausage facility harbouring 13 of them (Fig. S7B).

### Putative new species were identified through the reconstruction of metagenome-assembled genomes (MAGs) and facility-adapted strains of L. sakei and S. equorum were found by assembly-free strain level analyses

**Fig. 7:**
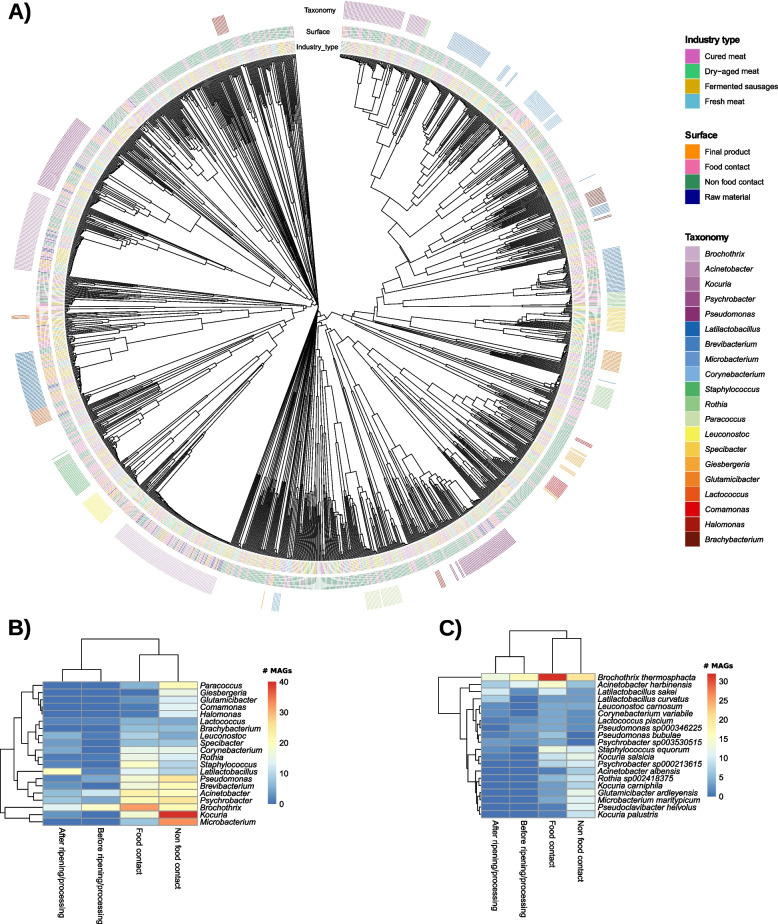
Reconstruction of metagenome-assembled genomes. **A** Phylogenetic plot of the 1421 MAGs obtained. Heatmap of **B** genera and **C** species distribution by sample type. Only the 20 most abundant taxa are shown in the heatmaps and indicated on the phylogenetic tree, sorted by number of MAGs per genus

A total of 1421 medium/high-quality MAGs were reconstructed (821 from NFC surfaces, 445 from FC surfaces, 104 from final products and 51 from raw materials/intermediate products before ripening), assigned to 273 genera and 254 species (Fig. 7A). The most abundant genera, with more than 40 MAGs obtained, were *Brochothrix* (*n* = 86), *Acinetobacter* (*n* = 62), *Kocuria* (*n* = 61), *Psychrobacter* (*n* = 59), *Pseudomonas* (*n* = 50), *Latilactobacillus* (*n* = 46), *Brevibacterium* (*n* = 41) and *Microbacterium* (*n* = 41), mainly found on FC and NFC surfaces (Fig. 7). MAGs belonging to *Latilactobacillus* were the most abundant on final products, while *Brochothrix* and *Acinetobacter* were mainly reconstructed from raw materials/intermediate products before ripening (Fig. 7B). The most reconstructed species were, *B. thermosphacta* (*n* = 86), *A. harbinensis* (*n* = 39), *L. sakei* (*n* = 26), *S. equorum* (*n* = 25) and *Kocuria salsicia* (*n* = 22). *B. thermosphacta* was the most frequently assembled in each sample type, while *A. harbinensis* was found predominantly in raw materials/intermediate products before ripening and *L. sakei* in final products. These species, together with *K. salsicia* and *S. equorum*, were the most reconstructed from FC surfaces, while NFC surfaces were more heterogeneous (Fig. 7C).

From the 612 high-quality MAGs (completeness > 90%), 274 correspond to 210 putative new species belonging to 132 different genera, 14 of which yet to be cultured. The genera for which we unravelled the widest unknown diversity were *Arthrobacter*, *Comamonas*, *Specibacter*, and *Giesbergeria* (4 new species reconstructed each); *Brevundimonas*, *Halomonas* and *Microbacterium* (*n* = 3); and *Brachybacterium*, *Brevibacterium*, *Corynebacterium*, Dietzia, *Kocuria*, and *Yaniella* (= 2). These putative new species were mostly found in NFC and FC surfaces, with 167 and 49 species respectively.

**Fig. 8:**
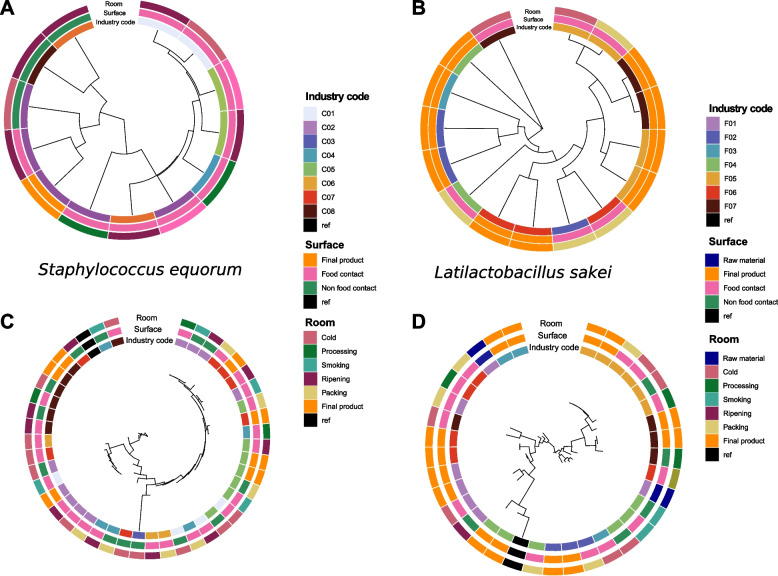
Phylogenetic trees of *S. equorum* and *L. sakei*. Phylogenetic trees obtained by **A**,**B** dRep processing of MAGs and **C**,**D** through StrainPhlan analysis. Only *S. equorum* from cured meat-producing facilities and *L. sakei* from fermented sausage-producing facilities are shown

Several plots based on ANI distances were built for each of the most abundant species in order to find species with strain specificity to a facility, with a high spread across the different samples within the same meat processing plant. Only *S. equorum* MAGs from cured meat-producing facilities and *L. sakei* from fermented sausage-producing plants presented certain level of clustering according to the facility (Fig. 8). However, the number of MAGs obtained was quite low. For that reason, similar strain-level analyses were done following an assembly-free approach, using StrainPhlan, and the same results were observed, with some level of clustering by facility for *S. equorum* and *L. sakei* on cured meat and fermented sausage-producing facilities, respectively (Fig. 8).

## Discussion

In the current study, we provide a detailed description of the core bacterial taxa that prevail in raw materials, end products and related processing environments in plants from different meat production sectors, including those processing fresh, dry-aged, cured and fermented meat products. The microbiome of raw materials slightly differed between pork and beef sources and the microbiome of end products was highly influenced by the microbiome of food processing environments, particularly of FC surfaces. In addition, evident changes in the taxonomic composition of the meat products were appreciated following processing/fermentation/ripening, which is in agreement with the observations of other authors [44, 45]. Nevertheless, 14 out of the 20 bacterial species with the highest relative abundance in the raw materials persisted also as the most abundant species in processing environments and/or in the final product, including species belonging to the genera *Pseudomonas*, *Brochothrix*, *Acinetobacter*, *Psychrobacter*, *Kocuria*, *Bacillus* and *Latilactobacillus*. Raw materials flow through different rooms inside each facility where they get in contact with different surfaces and environments, including tables and knives, which may pose an important source for bacterial contamination of meat. In addition, NFC surfaces within each room (e.g. floors, walls, drains) can also influence, and be influenced by, the meat microbiome. In these FC and NFC environments, microbes have the opportunity to adapt to the stress conditions prevailing during processing and establish niche-specific ecosystems [10]. The most common species in both surfaces were from the genus *Pseudomonas* and *Psychrobacter*, which have been previously recognized as common food spoilers. Specifically, in an oligotyping study, some oligotypes of *P. fragi* were the most abundant microbes in meat samples. However, other lower abundant *P. fragi* oligotypes highlighted the intra-species competition and the fact that distinct adaptation efficiencies are observed within different strains of the same species [46]. The co-occurrence of different strains of *P. fragi* and/or *Pseudomonas sp*. Lz4W could be the reason why no MAGs of this highly abundant species were obtained in the current work, due to the technical limitations of assembly and binning software. Remarkably, we found *P. fragi* and *Pseudomonas sp*. Lz4W as the only species of the core microbiota for all the surfaces and industry sectors, underlying the need for its control in the meat industry. *Pseudomonas sp*. Lz4W is a soil Antartic isolate initially classifed as *Pseudomonas syringae* Lz4W [47]. It is phylogenetically very close to *P. fragi* and it was proposed to be renamed as *Pseudomonas cryophila* Lz4W [48], as it is employed as a model system to understand the cold adaptation of bacteria [49]. Due to the disparity in the taxonomical assignation of this species and the possible absence in some of the commonly employed databases, there is limited information on its occurrence in previous metagenome studies.

Our findings agree with the findings of Stellato et al. [50], who found that *Psychrobacter* prevailed in environmental samples rather than on meat. The frequent identification of *P. fragi*, various *Psychrobacter spp.*, and *B. thermosphacta Psychrobacter* in environmental samples could be related to their well-known ability to form, or co-habit in, biofilms, as it has been previously observed in studies characterizing biofilms in meat processing environments [51]. On the contrary, *Leuconostoc* was not found among the taxa with the highest relative abundance in raw materials or surfaces, differently from what described in other studies in meat processing facilities [45, 52].

*L. sakei* was identified as the most dominant species in final products, especially in fermented sausages. *L. sakei* is well adapted to grow in this type of meat products, dominating the fermentation process and contributing to obtain sausages with optimal organoleptic characteristics, thus it is commonly used in starter cultures [53]. However, the majority of the fermented sausages included in this study were elaborated without the use of starter cultures. This suggests that *L. sakei*, in spite of not being added to the product, is widely established in the processing plants and easily proliferates in the product.

The higher relative abundance of *S. equorum* in final products than in raw materials of cured meat and fermented sausage production facilities can be related to the high abundance of this microorganism in FC surfaces. *S. equorum* has been previously isolated from meat products and the associated processing environments, suggesting a bidirectional influence between them [54].

The relative abundance of most members of the *Pseudomonas* genus suffered a significant decrease from raw materials to end products in fermented sausages, dry-cured meat and fresh meat product processing facilities. This could be explained by the co-exclusion relationship that Pothakos and colleagues [52] observed between this genus and members of the former *Lactobacillus* genus in processing environments and ingredients. Accordingly, in this study, the decrease of *Pseudomonas* was counterbalanced by an increase of *L. sakei* and *L. curvatus* along the processing of fermented sausages and fresh meat products.

The different meat products analysed in the current study are manufactured following quite different technological processes. On the one hand, in the elaboration of fermented sausages and cured meats, different spices, salt and/or additives are added and the product is subjected to a relatively long period (months) of ripening/curation. After these processes, the product has a relatively low water activity and, in the case of fermented sausages, low pH among other traits. Hence, a distinctive microbiota is present in the final products [55]. Gram-negative bacteria, such as *Pseudomonas*, are displaced by Gram-positive bacteria such as *Latilactobacillus* or *Staphylococcus*, which are generally more tolerant to low water activity and/or pH values. Indeed, some sensory attributes of these products are modulated by the activity of some Gram-positive bacteria, such as different LAB and gram-positive catalase positive cocci [56, 57].

Dry-aged beef is obtained after maturating the carcasses at very low temperatures (1–4 °C) and controlled relative humidity (70–90% RH) for some weeks or even months [58–60]. Some of the most prevalent microorganisms found in aged beef in other studies belong to *Pantoea*, *Pseudomonas* and *Streptococcus* [61]. In our study, *Pseudomonas* was found as a dominant taxon in the two end product samples, particularly *Pseudomonas* sp. Lz4W and *P. fragi*. Capouya and colleagues [62] also found a high abundance (65.2%) of *Pseudomonas* in dry-aged meat, and they assigned an operational taxonomic unit with 28.5% of relative abundance specifically to *P. fragi*.

On the contrary, fresh meat products do not experience a fermentation/curing/ripening process and, therefore, the dynamic changes in the microbiome from raw materials to end products should be less noticeable. Still, a shift from a dominance of *P. fragi* in raw materials to a high relative abundance of *L. sakei* and/or *B. thermosphacta* in the end products was observed. This variation could be possibly caused by the short period of storage (< 7 days) of final meat products in refrigeration chambers in the factory, which can induce physico-chemical and microbiome changes in the product. Our results obtained for fresh meat product facilities are consistent with the meta-analysis of Xu and colleagues [12], who commonly found *Pseudomonas*, *Acinetobacter* and *Psychrobacter* in surfaces, and *Brochothrix* in surfaces and meat products. They also found LAB to be dominant in final products while they are present in low abundances in surfaces and raw materials, and reported high relative abundance, especially, of *L. sakei* and, slightly, of *L. curvatus* and *L. carnosum* [12].

Several taxa potentially associated with safety concerns were detected in the current study, generally with low relative abundances. A previous study, in a beef production chain, reported that six pathogens (*Campylobacter*, *Clostridium*, *E. coli*, *Listeria monocytogenes*, *Salmonella enterica* and *S. aureus*) were drastically decreased from the arrival of the cattle to the feedlot to the commercial products [63]. In our study, *P. aeruginosa* was the most abundant bacterial among those associated with safety concerns, being more abundant in raw materials and NFC surfaces. *S. aureus* was the second most abundant. It was mainly found in FC surfaces and some final product samples. This corroborates previous findings where the incidence of *S. aureus* isolates was higher in raw materials than in FC and NFC surfaces, although no S. aureus was detected in the final products [64]. However, we cannot assume that the taxa possibly associated with safety concerns belong to pathogenic subtypes, considering that not all the strains from all these taxa pose a threat to public health. Nevertheless, the detection of genes associated with *S. aureus* enterotoxin production in some of the samples, even with low abundance, suggests the occurrence of some pathogenic strains from this species, although further isolate-based investigations into the virulence and ARG content of these particular strains would be required to completely assess their pathogenic potential.

The characterization of the resistome highlighted that the most abundant ARG were associated with resistance to tetracyclines, beta-lactams and aminoglycosides, which were already identified as the most abundant ARG families in other meat processing environments [8]. Nevertheless, we found some differences observing the less abundant ARG families: while in our samples fosfomycin ARG were the fourth most abundant ARG family and quinolones almost absent, Cobo-Díaz and colleagues [8] reported a higher prevalence of quinolones and a much lower abundance of fosfomycin ARG. In another study of a pork meat production chain, ARG linked to tetracyclines resulted to be the least abundant type of detected ARG [11]. Generally, the higher amount of ARG in surfaces than in raw materials and end products suggests that bacteria carrying ARG can survive and establish in surfaces, which then can act as a source of dispersal for these genes [8]. Similar results were obtained for *qac* genes, which were also highly abundant on surfaces, especially on FC surfaces. This result corroborates what Álvarez-Molina and colleagues [65] found in other FC surfaces from meat and dairy processing plants. These authors assigned the majority of the *qac* genes found to the *Staphylococcus* genus. In our study, FC surfaces from cured and fermented products particularly contained high abundance of *qac* genes and were also rich in *S. equorum*.

Yang and colleagues [63] found that, along a beef production chain, the VF detected belonged to four super families, namely *adhesion and invasion*, *secretion systems*, *toxins* and *iron acquisition*. Also, most of the VF in that study were specific to *E. coli*, but the majority of the VF were found in the cattle arriving at the feedlot and not in the commercial product [63]. In our case, very few genes associated with defined *E. coli* pathotypes were found, and further culture-dependent studies should be done to clarify this point. Furthermore, we focused on biofilm formation, adherence and exotoxin production-related genes, and found higher abundances of these genes in surfaces than in raw materials and final products, suggesting the importance of environmental surfaces as a reservoir of bacteria with potential pathogenic traits. Similar results of higher abundance of VF on environmental samples than on meat samples were previously reported on a pork production chain [66]. Biofilms entail a risk in the food industry, and although our results show the presence of biofilm-related genes in different meat processing plants, further research focussed in the biofilm formation behaviour of microbial communities is warranted to asses this issue.

The 612 high-quality MAGs obtained in meat samples included MAGs from 210 putative new species, belonging to 132 different genera, and represent an unprecedented resource of reconstructed bacterial genomes from meat, meat products and associated processing environments, which will be available, together with thousands of other bacterial genomes from non-meat based food chains, on the *curatedFoodMetagenomicData* (cFMD) database recently developed in the frame of the EU H2020 project MASTER [67]. Only a few studies related to meat metagenomes were available in the literature consulted. Some for *16S rRNA* amplicon sequencing, to study bacterial communities associated with meat spoilage [68] and from marinated and unmarinated broiler meat [69], and others for WMS on meat processing environments [8, 70]. However, to our knowledge, this is the first study reconstructing MAGs after applying WMS to meat industry samples. The identification in our results of a high diversity of putative new species demonstrates that there is an important microbial diversity still to be unravelled in meat processing environments, with functional roles also yet unexplored.

Remarkably, for two of the most relevant taxa in curing and fermentation processes (*S. equorum* and *L. sakei*, respectively), we found phylogenetic variation associated with the specific processing facility under study, which suggests that specific strains of these taxa may be selected in different meat processing plants, likely contributing to the peculiar sensorial traits of the end products produced in them. Indeed, Ferrocino and colleagues [71] previously described that *L. sakei* was positively correlated with different carbohydrate and amino acid metabolism pathways, deriving into branched-chain esters that are precursors of some aroma compounds. *S. equorum*, instead, was previously isolated from cured hams and further tested for its protease capacity [72]. Moreover, it has been previously shown that *Staphylococci* can influence the production of flavour substances due to their involvement in carbohydrate fermentation, amino acid conversion or lipid β-oxidation reactions [72].

## Conclusions

This study provides the most detailed metagenomics-based perspective up to now of the microbes that thrive in meat, meat products and associated environments opening avenues for future research activities to better understand their functionality and potential contribution to meat quality and safety. We have identified various genera as persistent from the raw materials to the processing surfaces and final products, while others were found as characteristic from certain sample categories or industry types. Many different ARGs and VFs have been identified, although further investigations should determine their relevance for food safety. Moreover, we have been able to recover hundreds of MAGs, including from several putative new species demonstrating that there is an important microbial diversity still to be unravelled in meat production systems.

## Supplementary Information

Supplementary Material 1: Supplementary Fig. 1. Beta-diversity by sample type. Principal Coordinates Analysis (PCoA) based on Bray–Curtis dissimilarity metrics of the bacterial communities of the different samples (A-D) taken in each meat industry sector. In fermented sausages facilities, the raw material category refers to meat batter and sausages before ripening.

Supplementary Material 2: Supplementary Fig. 2. Clustering of samples by taxonomy pattern. The relative abundances (%) of the forty most abundant species in material before ripening/processing/ageing, final products, food contact and non-food contact are represented for each industry type (dry-aged meat, cured meat, fermented sausages and fresh meat products facilities).

Supplementary Material 3: Supplementary Fig. 3. Taxonomy by animal species origin. Relative abundance of A) the 20 main species and B) some bacterial species possibly associated with safety concerns, found in raw materials or intermediate products before ripening coming from beef, pork or pork/beef mixtures.

Supplementary Material 4: Supplementary Fig. 4. Core microbiota shared among industry types for each sample category. A) Raw materials/intermediate products before ripening, B) Food contact surfaces, C) Non-food contact surfaces and D) Final products. The core microbiota is calculated as those species present on at least 90% of the samples and with relative abundance higher than 1% on at least 10% of the samples. All species corresponding to the same genus were coloured together. Only the 20 most relevant genera are represented.

Supplementary Material 5: Supplementary Fig. 5. ARG abundance. Amount of ARGs, expressed in CPM per bacterial marker, across the different meat industry sectors grouped by sample category.

Supplementary Material 6: Supplementary Fig. 6. *qac* genes abundance. Amount of *qac* genes, expressed in CPM per bacterial marker, across the different meat industry sectors grouped by sample category.

Supplementary Material 7: Supplementary Fig. 7. Virulence factors associated to A) *E. coli* pathotypes and B) S. aureus enterotoxins production. Heatmap represent total number of genes per sample detected.

Supplementary Material 8: Supplementary Table 1. Number of samples taken from different meat facilities, producing cured meat, dry-aged meat, fermented sausages and fresh meat products, respectively.* Raw material refers to the meat without any processing, apart from cutting; meat batter refers to the batter of minced meat with the rest of the ingredients added and mixed together before stuffing; meat before ripening/processing refers to the sausages after stuffing and before ripening; final product refers to the product once ready to be commercialized. ** Sausages kept in refrigeration chambers for < 1 week.

Supplementary Material 9: Supplementary Table 2. Genomes added to PlusPF database. GTDB-tk name is indicated if it is different from current name (according to NCBI). Kraken2:taxid column indicates the NCBI taxid for those genomes that initially failed to be correctly build in the database (fasta headers were change to add |kraken2:taxid|XXX to allow kraken2 to recognize the taxa of the genome). Finally, those genomes with missed FTP path were manually downloaded from NCBI by searching the Assemly Accession.

Supplementary Material 10: Supplementary Table 3. ResFinder database. Antimicrobial resistance genes clusters from ResFinder database used.

Supplementary Material 11: Supplementary Table 4. Virulence Factor genes selected as *E. coli* pathotype markers, according to Table A.1 from Koutsoumanis et al. [30]

Supplementary Material 12: Supplementary Table 5. ARGs found. List of antimicrobial resistance genes found on meat industry samples.

Supplementary Material 13: Supplementary Table 6. VF genes found. List of virulence factor genes found on meat industry samples.

## Data Availability

Raw reads generated from the sequencing and analysed during the current study were deposited into the Sequence Read Archive of the National Center of Biotechnology Information (NCBI) under the BioProject number PRJNA997800 and are available at https://www.ncbi.nlm.nih.gov/bioproject/?term=PRJNA997800. The code employed for raw reads filtering, assembly and binning into MAGs is available at https://github.com/SegataLab/MASTER-WP5-pipelines/tree/master/.

## Abbreviations

LAB: Lactic acid bacteria
FC: Food contact
NFC: Non-food contact
PBS: Phosphate-buffered saline
CPM: Counts per million
ARG: Antimicrobial resistance genes
SNPs: Single-nucleotide polymorphisms
ANI: Average nucleotide identity
NCBI: National Center of Biotechnology
PCoA: Principal coordinates analysis
VF: Virulence factor
MAG: Metagenome-assembled genome
RH: Relative humidity
