# Supplementary material for: In-depth characterization of food and environmental microbiomes across different meat processing plants
Source: Microbiome. 2024 Oct 15;12:199. doi: 10.1186/s40168-024-01856-3 (PMC11481301; doi:10.1186/s40168-024-01856-3)
Supplement: Supplementary file 2 — Supplementary Material 1: Supplementary Fig. 1. Beta-diversity by sample type. Principal Coordinates Analysis (PCoA) based on Bray–Curtis dissimilarity metrics of the bacterial communities of the different samples (A-D) taken in each meat industry sector. In fermented sausages facilities, the raw material category refers to meat batter and sausages before ripening. [file 40168_2024_1856_MOESM1_ESM.pdf]

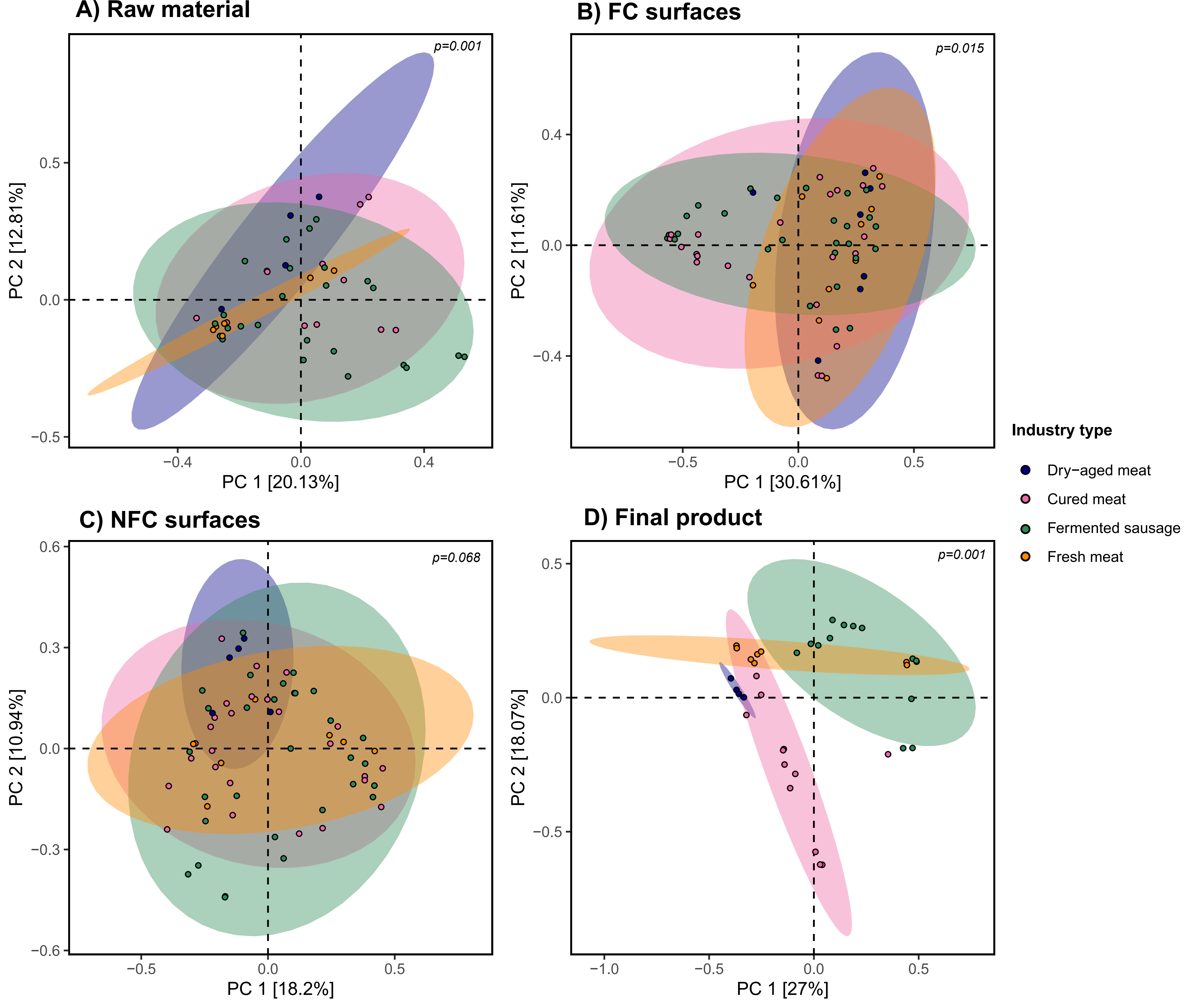
A) Raw material
p=0.001
PC 2 [12.81%]
PC 1 [20.13%]
0.5
0.0
−0.5
−0.4
0.0
0.4
B) FC surfaces
p=0.015
PC 2 [11.61%]
PC 1 [30.61%]
0.4
0.0
−0.4
−0.5
0.0
0.5
C) NFC surfaces
p=0.068
PC 2 [10.94%]
PC 1 [18.2%]
0.6
0.3
0.0
−0.3
−0.6
−0.5
0.0
0.5
D) Final product
p=0.001
PC 2 [18.07%]
PC 1 [27%]
0.5
0.0
−0.5
−1.0
−0.5
0.0
0.5
Industry type
Dry−aged meat
Cured meat
Fermented sausage
Fresh meat
