# Supplementary figures and images for: In-depth characterization of food and environmental microbiomes across different meat processing plants
Source: Microbiome. 2024 Oct 15;12:199. doi: 10.1186/s40168-024-01856-3 (PMC11481301; doi:10.1186/s40168-024-01856-3)

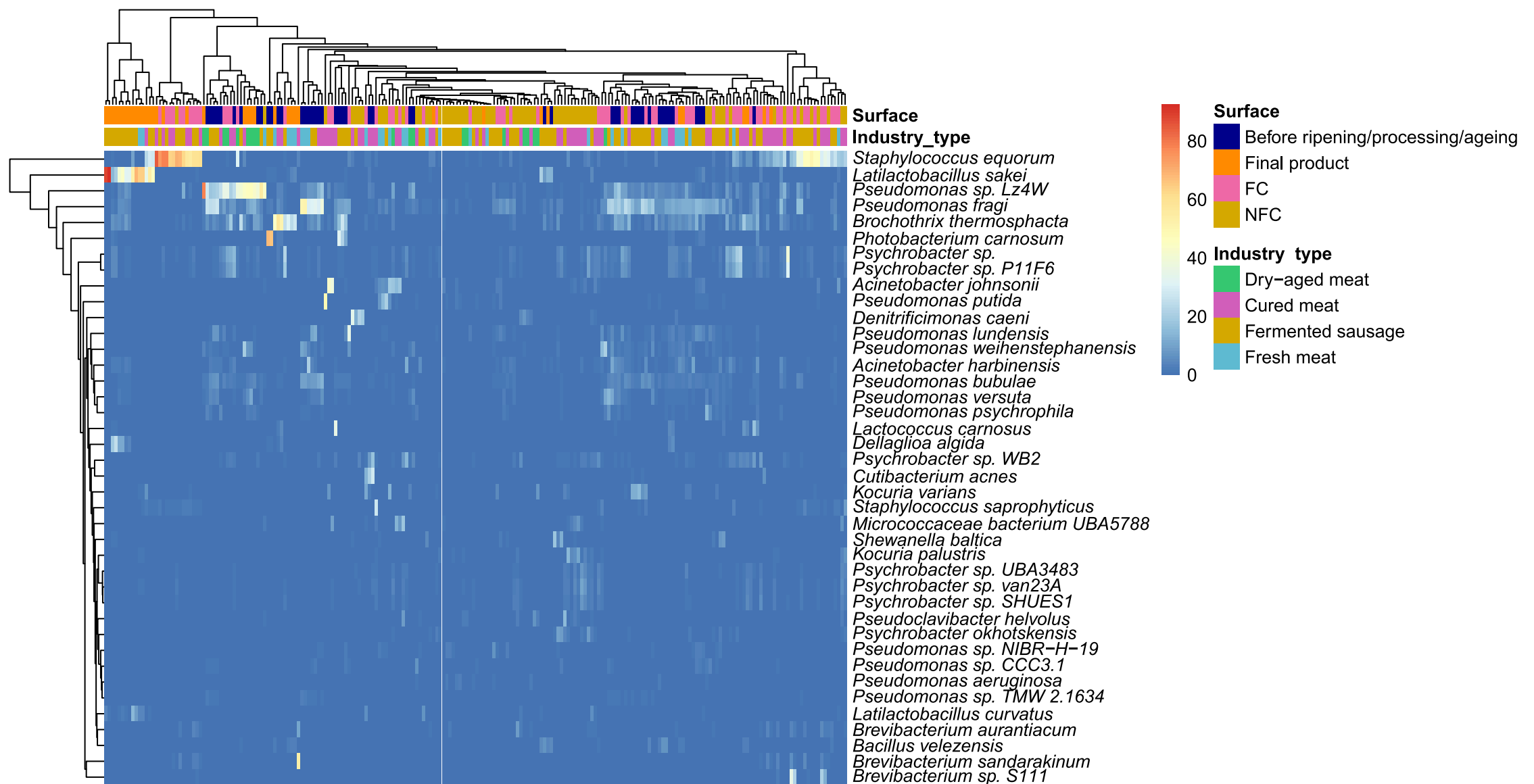

Supplement: Supplementary file 3 — Supplementary Material 2: Supplementary Fig. 2. Clustering of samples by taxonomy pattern. The relative abundances (%) of the forty most abundant species in material before ripening/processing/ageing, final products, food contact and non-food contact are represented for each industry type (dry-aged meat, cured meat, fermented sausages and fresh meat products facilities). [file 40168_2024_1856_MOESM2_ESM.pdf]

**A**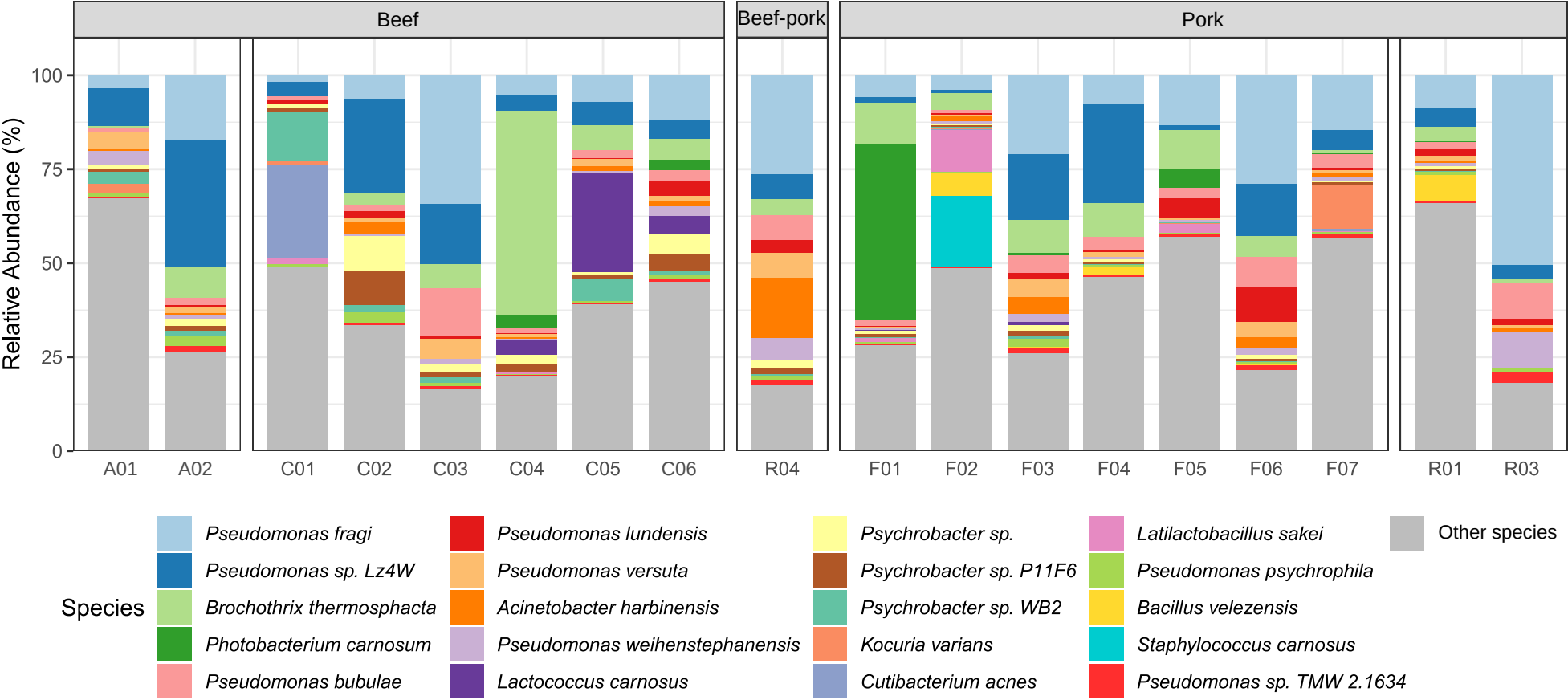**B**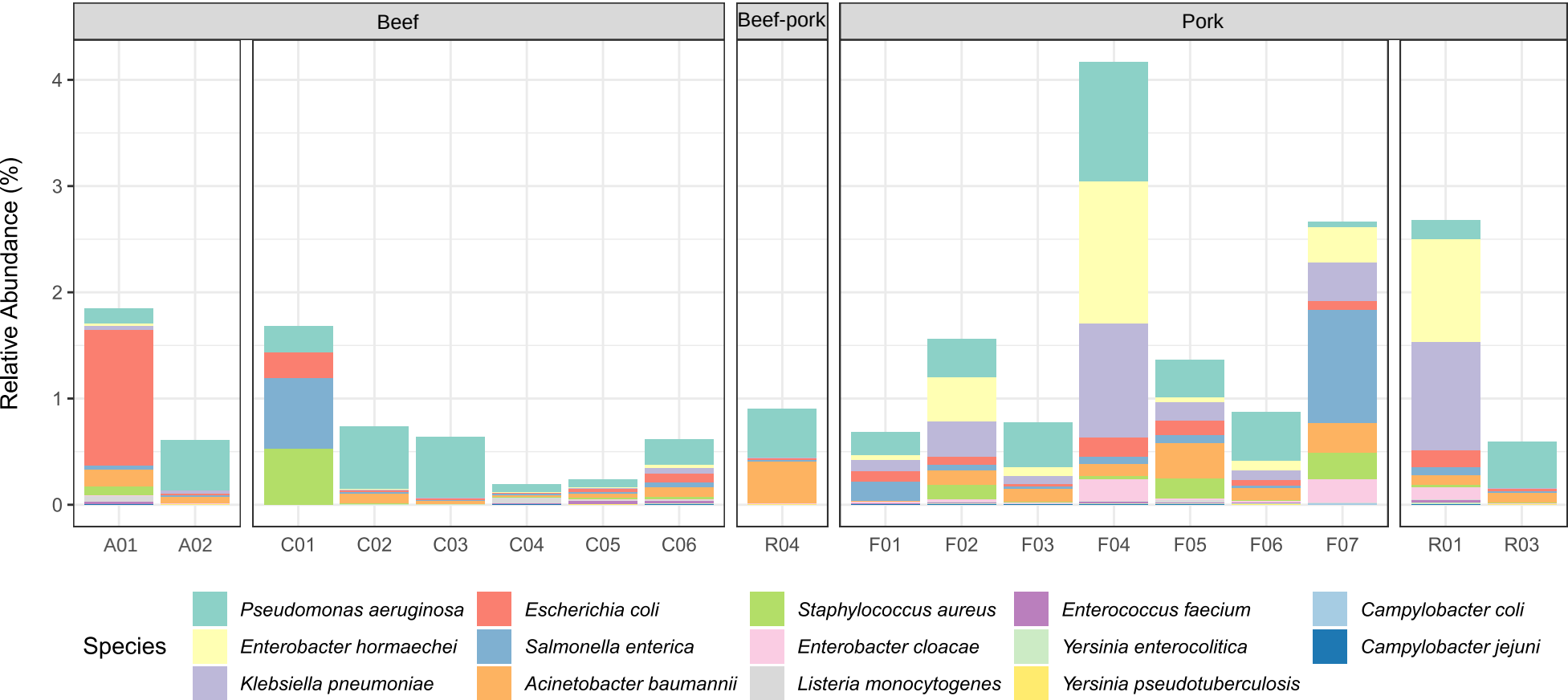

Supplement: Supplementary file 4 — Supplementary Material 3: Supplementary Fig. 3. Taxonomy by animal species origin. Relative abundance of A) the 20 main species and B) some bacterial species possibly associated with safety concerns, found in raw materials or intermediate products before ripening coming from beef, pork or pork/beef mixtures. [file 40168_2024_1856_MOESM3_ESM.pdf]

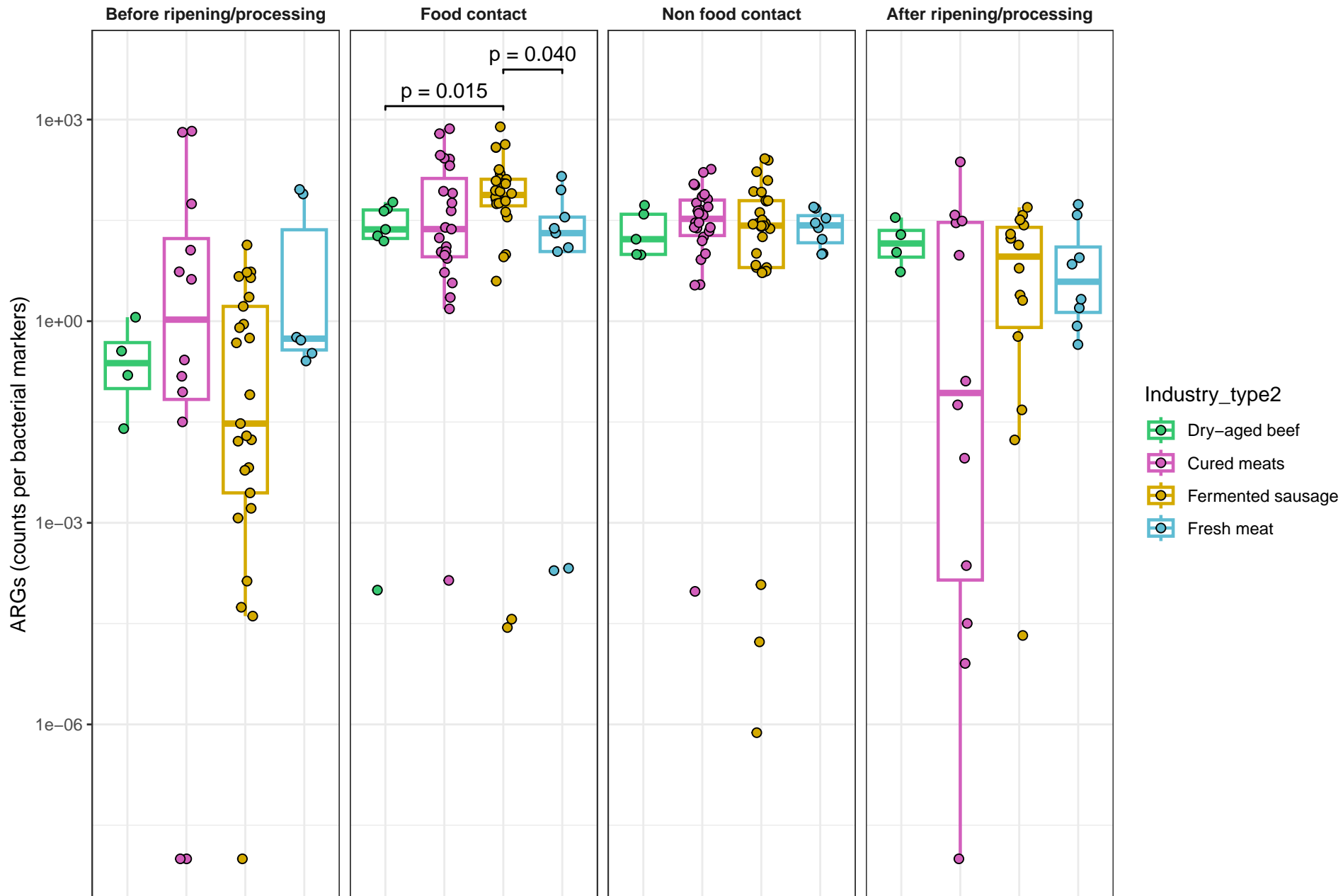

Supplement: Supplementary file 6 — Supplementary Material 5: Supplementary Fig. 5. ARG abundance. Amount of ARGs, expressed in CPM per bacterial marker, across the different meat industry sectors grouped by sample category. [file 40168_2024_1856_MOESM5_ESM.pdf]

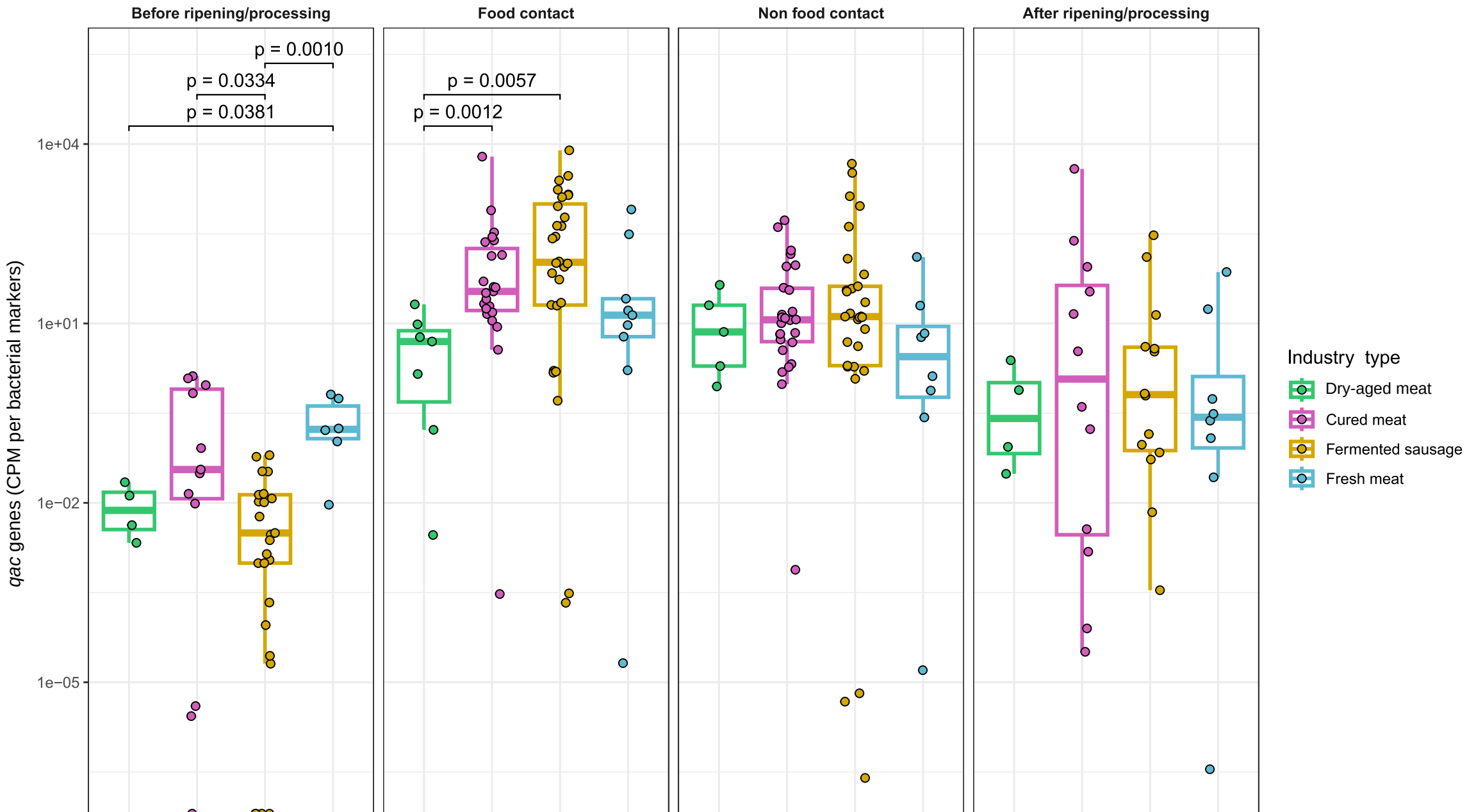

Supplement: Supplementary file 7 — Supplementary Material 6: Supplementary Fig. 6. qac genes abundance. Amount of qac genes, expressed in CPM per bacterial marker, across the different meat industry sectors grouped by sample category. [file 40168_2024_1856_MOESM6_ESM.pdf]

**A**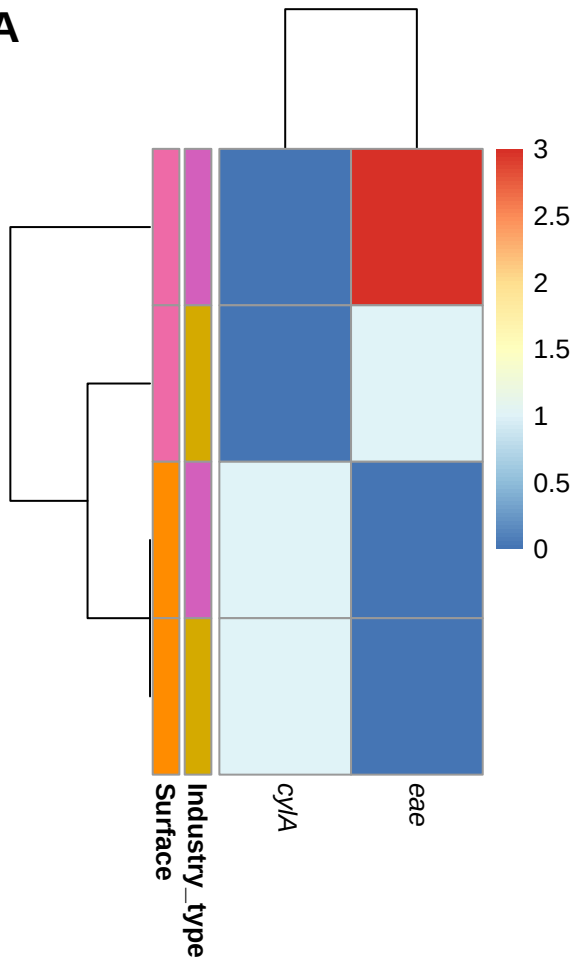**B**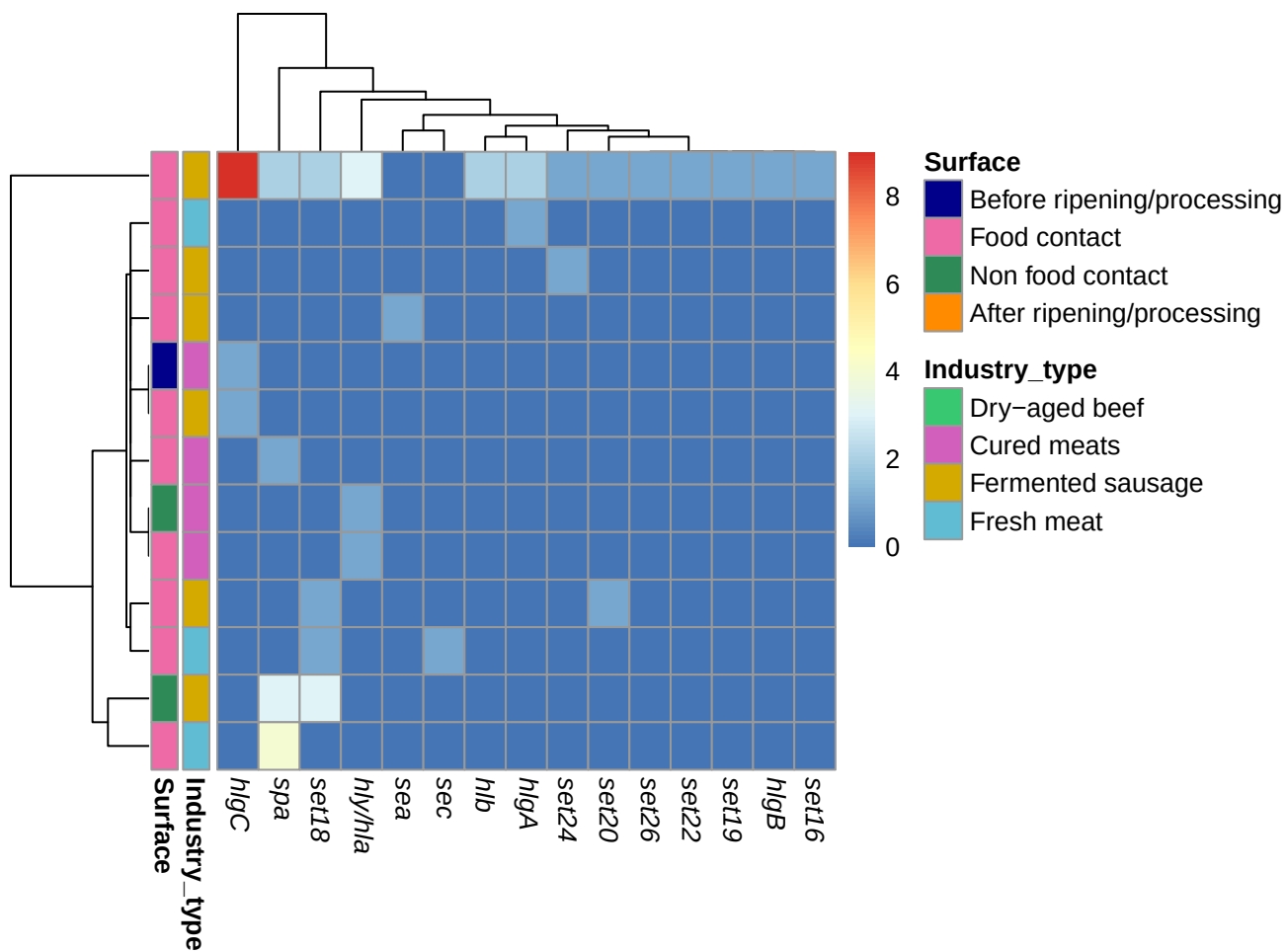

Supplement: Supplementary file 8 — Supplementary Material 7: Supplementary Fig. 7. Virulence factors associated to A) E. coli pathotypes and B) S. aureus enterotoxins production. Heatmap represent total number of genes per sample detected. [file 40168_2024_1856_MOESM7_ESM.pdf]
