# Supplementary material for: In-depth characterization of food and environmental microbiomes across different meat processing plants
Source: Microbiome. 2024 Oct 15;12:199. doi: 10.1186/s40168-024-01856-3 (PMC11481301; doi:10.1186/s40168-024-01856-3)
Supplement: Supplementary file 5 — Supplementary Material 4: Supplementary Fig. 4. Core microbiota shared among industry types for each sample category. A) Raw materials/intermediate products before ripening, B) Food contact surfaces, C) Non-food contact surfaces and D) Final products. The core microbiota is calculated as those species present on at least 90% of the samples and with relative abundance higher than 1% on at least 10% of the samples. All species corresponding to the same genus were coloured together. Only the 20 most relevant genera are represented. [file 40168_2024_1856_MOESM4_ESM.pdf]

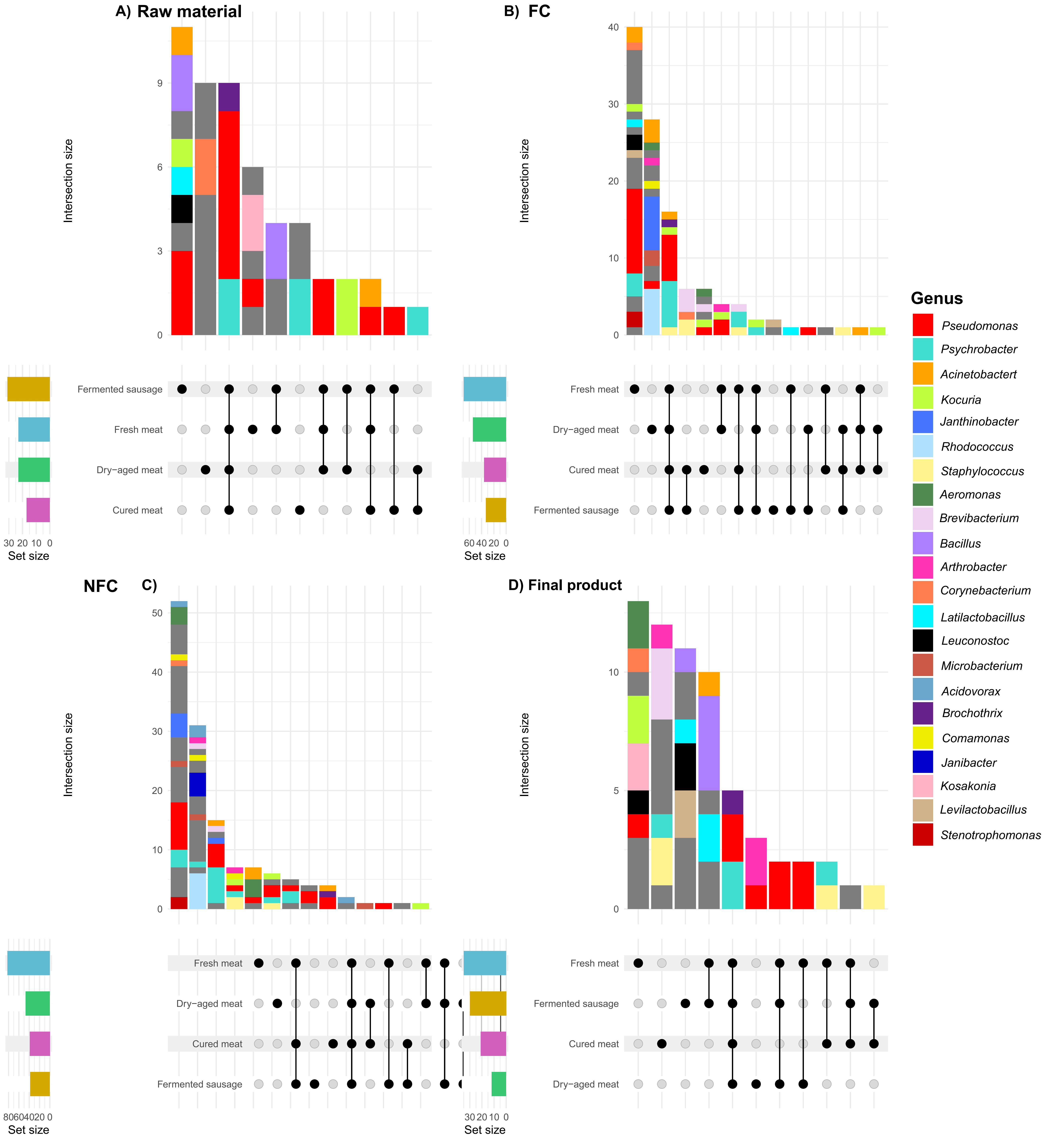

A) Raw material
B) FC
NFC
C)
D) Final product
Intersection size
Set size
Fresh meat
Dry−aged meat
Cured meat
Fermented sausage
Genus
Pseudomonas
Psychrobacter
Acinetobactert
Kocuria
Janthinobacter
Rhodococcus
Staphylococcus
Aeromonas
Brevibacterium
Bacillus
Arthrobacter
Corynebacterium
Latilactobacillus
Leuconostoc
Microbacterium
Acidovorax
Brochothrix
Comamonas
Janibacter
Kosakonia
Levilactobacillus
Stenotrophomonas
